# Influence of a rural Longitudinal Integrated Clerkship on medical graduates’ geographic and specialty decisions: a constructivist grounded theory study

**DOI:** 10.1186/s12909-024-05793-5

**Published:** 2024-07-24

**Authors:** Jessica Beattie, Marley Binder, Hannah Beks, Lara Fuller

**Affiliations:** 1 School of Medicine, Deakin University, Rural Community Clinical School, PO Box 713, Colac, Vic 3250 Australia; 2 School of Medicine, Department of Rural Health, Deakin University Princess Hwy, Warrnambool, 3280 Australia

## Abstract

**Background:**

Like many countries, Australia is suffering from a longstanding and persistent medical workforce maldistribution with fewer doctors per capita in rural locations and a trend towards sub-specialisation. Longitudinal Integrated Clerkships (LIC), a medical education model, are more likely than other clerkship models to produce graduates who work rurally, in communities of increasing remoteness and in primary care. While this quantitative data is essential, there has been a dearth of program-specific evidence explaining this phenomenon.

**Methods:**

To address this knowledge gap, a constructivist qualitative grounded theory approach was employed to identify how the Deakin University comprehensive rural LIC influences graduates’ (2011–2020) career decisions in terms of both medical specialty and geographic practice location.

**Results:**

Thirty-nine graduates participated in qualitative interviews. The Rural LIC Career Decision Making Framework was developed, postulating that an alignment of personal and program factors under the central concept of ‘choosing to participate’ can influence graduates’ geographic and specialist career decisions, both individually and symbiotically. Once embedded in the clerkship, participation was augmented by the concepts of learning design affordance and learning in place, providing the participants with longitudinal opportunities to experience and compare medical disciplines in an integrated manner.

**Conclusions:**

The developed framework presents contextual elements of the program that were deemed influential on graduates’ subsequent career decisions. The alignment of these elements with the program’s mission statement has the capacity to enhance the program’s rural workforce goals. Regardless of graduates’ willingness to participate in the program, a transformation occurred. Transformation occurs through reflection, either challenging or confirming the graduate’s pre-conceived ideas about career decisions and in turn influencing professional identity formation.

## Introduction

Like many countries, Australia is suffering from a longstanding and persistent medical workforce maldistribution [1]. This is evidenced by fewer doctors per capita in rural locations and a trend away from careers in primary care towards sub-specialisation [2, 3]. Combined, these factors have a detrimental effect on rural health, particularly as primary care is the linchpin of the medical workforce in these communities providing care across not only primary care but also emergency departments and inpatient settings [4]. Longitudinal Integrated Clerkships (LIC), a medical education model, were initially developed as a way of training medical students in smaller rural communities with a founding aim to encourage eventual practice in similar communities [5, 6]. This aspirational goal is being realized, with rural LIC graduates more likely than graduates of other clerkships (including rural block rotations) to work rurally, in communities of increasing remoteness, and in primary care [7–10]. The way medical graduates make their career choices has been described as a complex process that includes a range of intrinsic and extrinsic factors such as lifestyle choices and health system structures [11–13]. Less attention has been paid to elements of undergraduate medical training that may be formative in this decision-making process.

The pedagogy of an LIC differs from traditional block rotations in terms of structure and setting [5, 14–16]. Rural LICs are often located in smaller rural communities with clinical attachments to both a general practice and hospital inpatient setting [5]. A core LIC element is the concept of ‘continuity’ which is facilitated by longitudinal attachments allowing students to develop long-term relationships with supervisors, health care teams, and patients [5, 14–16]. LIC students learn the curriculum in an integrated parallel manner, in contrast to the time-limited sequential disciplines specific to traditional block rotations [5, 17].

While quantitative LIC workforce data is essential for evaluating program outcomes there has been a dearth of program-specific evidence explaining why rural LIC graduates are more likely to work rurally and in primary care compared to medical graduates from other clerkship models [8, 18]. Brown et al. (2021) conducted a scoping review on professional identity formation in LICs (both urban and rural) and suggested further information is required about the contextual elements that facilitate how LICs work to provide an understanding of the mechanisms that influence graduates’ career decisions [18]. Furthermore, work is required to understand LIC graduates’ career choices retrospectively, by engaging with graduates once they are qualified doctors who have made specialty decisions, as many studies have focused on the prospective opinions and intentions of students and junior doctors [11, 18, 19].

The phenomenon of interest is to explore how a comprehensive rural LIC program influenced graduates’ career decisions in terms of both medical specialty and geographic practice location. A constructivist grounded theory methodology was employed to answer the research question and develop a conceptual framework depicting elements of the clerkship that were influential in this process.

## Methods

### Study design

This was a qualitative constructivist grounded theory project. This was determined to be the most appropriate grounded theory methodology as (i) it acknowledges that the relationship between the researcher and participant forms the foundation of the data collection which is essentially co-constructed between the two parties (ii) it is deemed an appropriate methodology for social justice inquiry such as the equitable distribution of medical resources and (iii) it allows for the explanation of a phenomenon e.g. ‘what is going on?’ rather than to simply explore and describe it [20].

### Setting

Deakin University’s Doctor of Medicine (MD) degree (formally Bachelor of Medicine/Bachelor of Surgery) commenced in 2008. The MD is a four-year graduate entry program distributed across both metropolitan and rural locations predominately located in western Victoria, Australia. According to Australia’s Modified Monash Model (MMM) geographical remoteness classification system, MD course delivery locations include MM1 (Metropolitan areas), MM2 (Regional centers), MM3 (Large rural towns), MM4 (Medium rural towns) and MM5 (Small rural towns) [21].

**Fig. 1:**
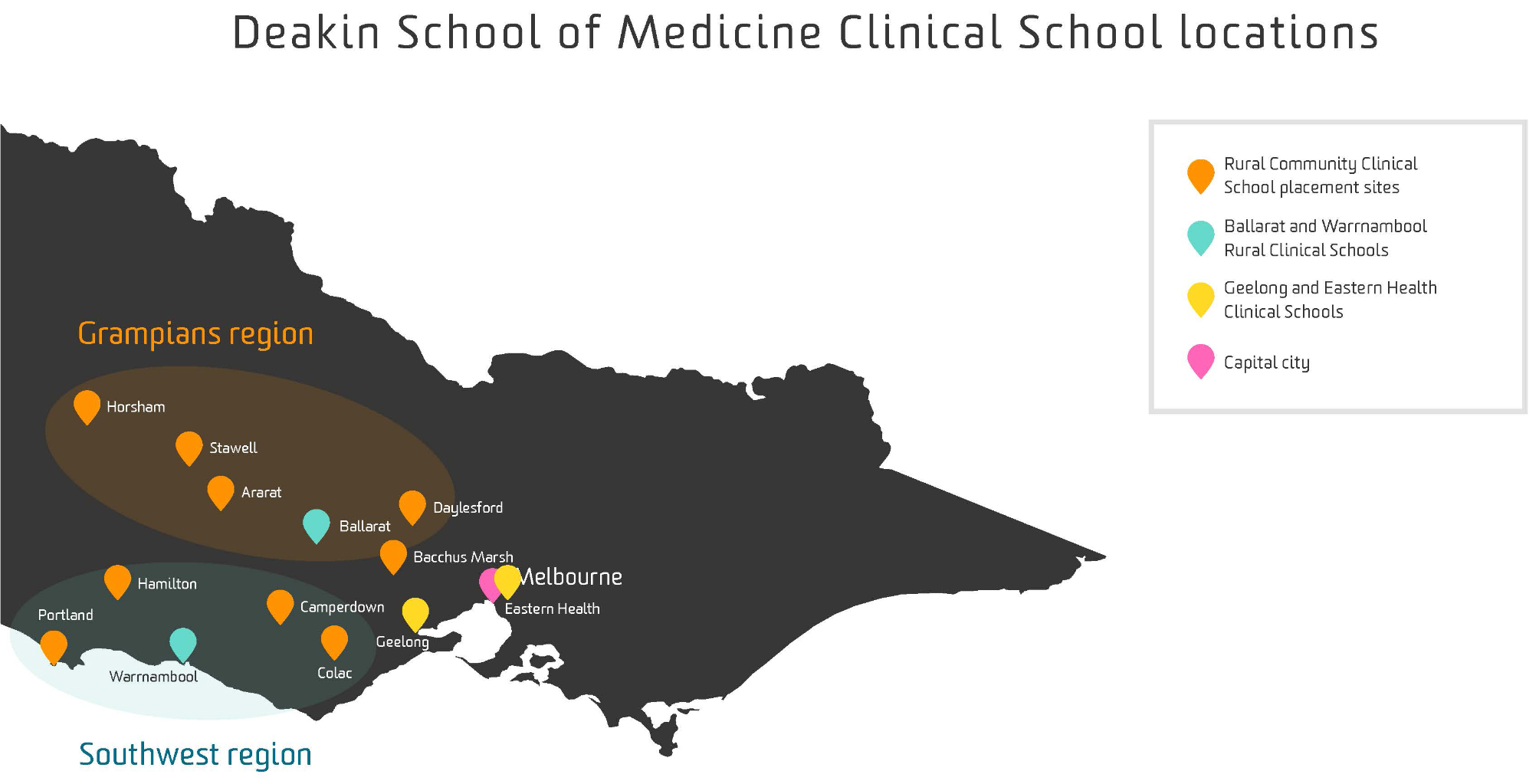
Deakin University Clinical School Map. © Deakin University. Reproduced with permission

The first two pre-clinical years (*Foundations of Medicine*) are based in Geelong (MM1). In years three and four, students undertake clinical training (*Professional Practice of Medicine*) at one of five clinical schools; Geelong, Eastern Health (MM1), Ballarat (MM2), Warrnambool (MM3), or the LIC program - the Rural Community Clinical School (RCCS), formally known as the IMMERSE program before 2014 (MM 3–5) (Fig. 1).

The RCCS LIC which admits approximately 20 students per year, operates across the Grampians and Southwest regions of Victoria during the penultimate year (year three) of the MD. The selection method is by a preferencing system where in year two students nominate their clinical school preferences. The program admits students with a range of preferences from first to fifth. Specific towns are later allocated through student preferencing and an interview process. Predominately, students are allocated to towns in groups of two to four students.

Students are attached to a general practice and local rural health service with a general practitioner (GP) as their primary supervisor.

In year four students relocate to one of the other clinical schools.

### Researchers reflexive statement

There were four researchers involved in this study with different backgrounds and professions but a common interest in medical education and equitable distribution of the medical workforce. As we employed constructivist grounded theory, we believe that our backgrounds, experiences, professions, beliefs, and interests informed the development of the research question, interview process, data analysis, and theory generation. JB is a rural health researcher experienced in qualitative research who is employed in the LIC and resides rurally within the LIC training footprint. LF is an academic general practitioner and Clinical School Director of the LIC program at Deakin University who is involved in the education of LIC students. MB and HB are rural researchers experienced in qualitative research projects who live rurally within the LIC training footprint.

Reflexivity and the array of researchers’ experiences and skills were employed to interpret and find meaning within this rich data set. Frequent discussions were held throughout the data collection and analysis process, particularly between JB and MB. HB and LF provided support during this process and through the development of high-level concepts and theory development.

### Participants

Participants were Deakin University medical graduates (2011–2020) who had undertaken the LIC. An invitation to participate in the study was distributed via a recruitment text message from an RCCS professional staff member. Interested participants were instructed to follow the registration link and submit their details via a Qualtrics survey [22] indicating they (i) had read the plain language statement outlining the research purpose and participant requirements and (ii) were willing to be contacted by a researcher to arrange a suitable interview time. Participants’ geographic work location was also recorded.

### Sampling

**Fig. 2:**
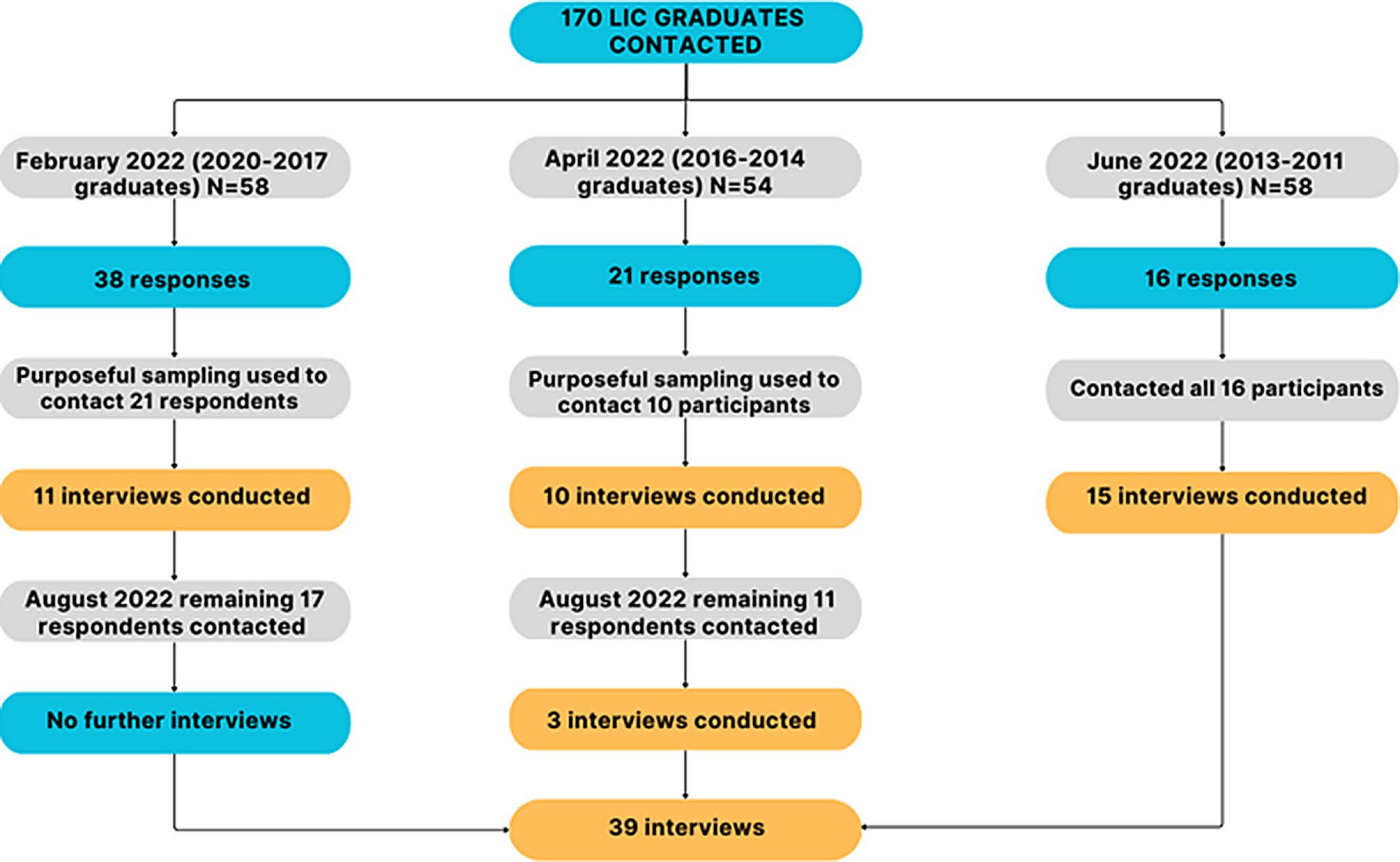
Participant recruitment flow chart. LIC graduates refers to Longitudinal Integrated Clerkship participants. Purposeful sampling refers to recruiting a diverse sample of participants

Participants were recruited in three stages: stage one 2017–2020 graduates, stage two 2014–2016 graduates and stage three 2011–2013 graduates (Fig. 2). Purposeful sampling was initially used to contact interested graduates to ensure there was a range of geographic work locations represented. Some graduates who initially expressed interest in participating in the study were not interviewed as they did not respond to researchers’ requests for an interview time. The staged recruitment process allowed for an iterative data collection and analysis process that supported theoretical sampling, the elaboration and refinement of concepts, and theory generation [20].

Participants received a $150 Mastercard gift card as reimbursement for participation in the study.

### Data collection and analysis

Interviews were conducted by researchers JB and MB. Verbal consent was gained from participants and audio-recorded before the commencement of the interview. A semi-structured interview guide and associated probes were initially developed to guide the interview process (Table 1). The guide was subsequently amended and reviewed through the data collection and analysis process to integrate lines of inquiry aligned with theory generation. Interviews were conducted over the telephone, audio recorded, transcribed verbatim, and de-identified. Interview duration was 20–53 min with a mean time of 33 min. Before data analyses a copy of the transcribed interviews was sent to participants allowing them to add or redact information.

**Table 1:** Indicative interview guide

Question	Potential Prompts (following lines of inquiry)
Could you tell me about your journey to become a medical professional?	Why pursue medicine? Were there any enablers or barriers?
Could I ask you about your reflections on your LIC year?	What disciplines did you get the most experience in? Did you spend more time in any particular setting? How did this influence your career? Reflecting on the LIC year do you think it influenced your career decisions? How etc?
Are there other factors that have informed your decision to practice in X (insert where they are and medical specialty)?	What were your professional influences? What were your personal influences?

Interview transcripts were uploaded to a qualitative software package QSR NVivo for Windows, version 12 (Lumivero) to supplement data analysis [23]. Researchers JB and MB individually listened to, read, and coded each interview. Memo writing was constantly used to record informal ideas about the data, codes, and theoretical categories [20].

Data collection and analyses occurred concurrently, with each process informing the other. This constant comparative method transversed all stages of data analyses. For example, comparing data to data, fracturing and refining codes allowing for the development of further lines of inquiry aligned with theory development [20]. Researchers JB and MB met frequently to discuss the initial coding, and to identify areas to focus on during the iterative data collection process.

Coding commenced with initial line-by-line coding where data was ‘fractured’ and ascribed open codes, describing actions and processes associated with ‘what was happening’ in the data. The next stage of coding was intermediate coding where the line-by-line codes were viewed together, compared, analyzed, and conceptualized to determine which codes made the most analytic sense in categorizing the data [20]. Finally, advanced theoretical coding was employed to build a theory. This involved the entire research team, discussing and agreeing on the analytical properties of the theory, ensuring it explained the phenomenon clearly.

Demographic data was collected via a quantitative online survey before each interview to ensure there was a range of participants and to supplement qualitative analyses. Data collected included: gender, age, graduation year, rural background, current geographic work location, medical specialty, and 4th year clinical school location.

## Results

The results generated in the study informed the development of a conceptual framework illustrating how the rural LIC influenced graduates’ geographic and specialty career decisions.

### Quantitative results

A total of 39 LIC graduates participated in the study. Briefly, females accounted for 53.8% of participants, 43.6% were from a rural background, 38.5% were working rurally and 89.7% had either completed their medical specialty training or had joined a training pathway (Table 2).

**Table 2:** LIC participants’ demographic details

Characteristic	Percentage (*n* = 39)
Gender Male Female	46.2% (*n* = 18) 53.8% (*n* = 21)
Age groups	
*<*30	12.8% (*n* = 5)
30–34	46.2% (*n* = 18)
35–39	30.8% (*n* = 12)
> 40	10.2% (*n* = 4)
**Rural background**	
Yes	43.6% (*n* = 17)
No	56.4% (*n* = 22)
**Principal place of practice**	
Rural	38.5% (*n* = 15)
Metropolitan	61.5% (*n* = 24)
**Principal place of practice by Monash Modified Model**	
MM1	61.5% (*n* = 24)
MM2	10.3% (*n* = 4)
MM3	12.8% (*n* = 5)
MM4	10.3% (*n* = 4)
MM5	5.1% (*n* = 2)
MM6	0
MM7	0
**Clinical training pathway**	
LIC and rural 4th year	35.9% (*n* = 14)
LIC and metropolitan 4th year	64.1% (*n* = 25)
**Graduation year**	
2011–2013	38.5% (*n* = 15)
2014–2016	33.3% (*n* = 13)
2017–2020	28.2% (*n* = 11)
**Joined a vocational/medical specialty training pathway or a qualified medical specialist?**	
Yes	89.7% (*n* = 35)
No	10.3% (*n* = 4)
**Vocational pathways**	
General Practice	38.5% (*n* = 15)
Physician training	28.2% (*n* = 11)
Anaesthetics	12.8% (*n* = 5)
Other*	10.2% (*n* = 4)

*A combination of medical specialties was grouped as reporting individually was deemed to have the potential to identify participants

MM: Modified Monash Model. Australian geographic measure of remoteness

LIC: Longitudinal Integrated Clerkship

### Theoretical Framework

**Fig. 3:**
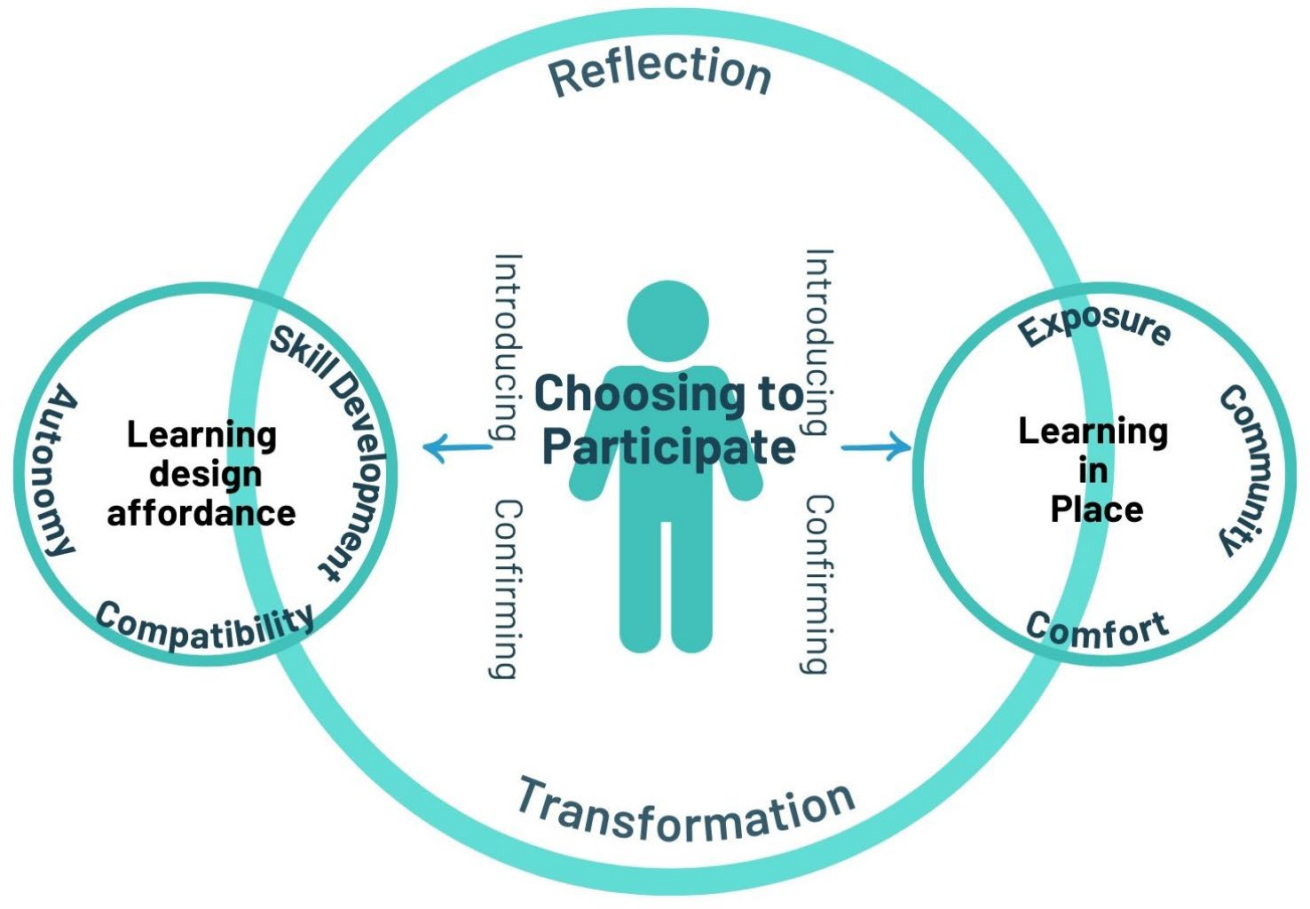
The Rural LIC Career Decision-Making Framework

This Rural LIC Career Decision-Making Framework is focused on the rural LIC program elements that influence graduates’ career decisions, postulating that an alignment of personal and program factors under the central concept of ‘choosing to participate’ has the capacity to influence graduates’ geographic and specialist career decisions, both individually and symbiotically (Fig. 3). Elements of the framework are described in the following qualitative results, with participants’ quotes included to illustrate meaning.

### Choosing to participate

Clinical school allocation was conducted via a preferencing system and as such there was variance in participants’ self-selection into the program. *Choosing to participate* nominally encompassed two groups of graduates; those who purposely chose participation in the program (self-selection) and those who did not choose but were allocated to the RCCS. This was reflected in the concepts of *introducing (latter group)* and *confirming (former group)*. Once embedded in the clerkship, participation was augmented by the concepts of *learning design affordance* and *learning in place*, providing the participants with longitudinal opportunities to experience and compare medical disciplines in an integrated manner.

## Introducing

Regardless of the level of self-selection, participants predominately reflected on their experience positively, stating the LIC was a formative training year that not only introduced them to the clinical environment but provided a strong foundation for their learning and career continuum. They were introduced to rural living, rural medicine, general practice, and various medical specialties through the integrated manner of program delivery.

Some participants reflected that if they had not participated in the program and had completed all their training in a metropolitan setting, they would never have considered and been introduced to how both their personal and professional needs could be met in the rural environment. This culminated in the merging of personal and professional factors such as the type of doctor they aspired to be, the community they wished to practice in, and lifestyle aspects such as access to the outdoor environment and the affordability of living rurally.

I feel like if I had just stayed in X [metropolitan site] or something like that then we’d probably would’ve just stayed in the one place, I don’t think we (partner) would’ve made that, that jump (to work rural) without a push to do it (GP registrar, rural practice).

### Confirming

Participation in the program provided the opportunity to reflect and confirm the graduate’s intent to work rurally. This was often associated with growing up in a rural environment and having an intent to practice in a similar location upon graduation. Confirming was also evident in participants who had a pre-existing intent to work in general practice with the experience meeting their expectations and consolidating their pursuit of this pathway.

And it (participation in LIC) just sort of solidified what I thought was already going to be my preference and it just was kind of sealed the deal really, I didn’t… even consider applying for metro places in my intern year or have never even thought of working in metro really (Psychiatrist, rural practice).

For others, participation confirmed that rural living/medicine did not align with their personal and professional needs. Personal concerns were distance from family and friends and access to services such as education, and healthcare. The frequency of on-call work they perceived that rural clinicians undertake was a professional deterrent.

The supervisors in my town were on call quite a lot. So that lifestyle, I think, wouldn’t suit me (General Physician, metropolitan practice).

### Learning design affordance

Learning design affordance, and how the students’ learning was structured were influential in facilitating career decisions. The central LIC elements of continuity and integration afforded participants autonomy and a range of opportunities that enabled them to actively participate in patient care, developing skills and facilitating real-time discovery and comparison of the type of medical practice that was compatible with their personal and professional needs.

### Autonomy

As medical disciplines within the curriculum were learned in an integrated manner, participants had a high level of autonomy to self-direct and seek out their own learning opportunities. Participants’ autonomy progressively grew throughout the year as they gained an innate understanding and feeling of safety within the confines of the program’s structure, gaining agency for in-depth self-exploration of a range of clinical settings. This enabled the comparison of medical disciplines in real-time with participants reflecting that they were drawn to particular clinical areas which they have often eventually pursued as a specialty.

In RCCS just having access to those specialties earlier and then actually being able to get a lot more time in the disciplines that you actually are interested in, so, of course, the more metro students weren’t able to choose when and where they’d actually go in the hospital on any given day… and so that meant for me I was able to spend more time in the emergency department, a little bit more time in theatre, doing things that I found more interesting (Anesthetist, rural practice).

The structure of the program allowed students to encounter undifferentiated patients providing a safe level of autonomy to take patient histories, develop clinical reasoning skills and present differential diagnoses and management plans to clinicians. This autonomy was contrasted against returning to a block rotation in year four where there was a perception of less access to undifferentiated patients and a transition back into an observer role. For example, one student reflected that if their only clinical experience in general practice had been the time-limited fourth-year rotation where they described being an observer, they would not have understood the breadth of general practice and suggested they would have pursued another specialty.

And I didn’t have a great experience at all (GP block rotation). So, I felt that maybe my career choice would have been different if that was my only experience in general practice…I just felt like it was a bit of a waste of a placement like I was just observing (GP, rural practice).

### Compatibility

The longitudinal attachment allowed participants to develop continuous relationships with clinicians who became mentors and role models. Participants actively sought out and spent extended periods with clinicians for a variety of reasons such as the time and support they gave them, teaching acumen, approachability, admiration for their model of practice, and compatibility of their personality and values with their own or those they aspired to develop. Role models/mentors were not only participants allocated primary GP supervisors but came from a variety of medical specialties including physicians, surgeons, and anesthetists.

A couple of things. I was out in X (LIC location). There was (an) anesthetist slash ICU I guess he looked after the ICU at X (rural) hospital, the calm attitude and most of the anesthetists I’ve met are pretty unfazed by most things. Just that kind of unflappable attitude really resonated with me. (Anesthetist, metropolitan practice)

A realistic understanding of the intersection of clinicians’ professional and personal lives was described as an invaluable insight into their lifestyle and was credited with encouraging participants to follow similar paths. There was also an idealization of clinicians’ lives that was gleaned by socialization at their homes.

### Skill development

Throughout the year participants developed clinical, personal, and professional skills through hands-on learning opportunities afforded by the relationships they built with clinicians, patients, and medical teams. Development of these clinical and communication skills often pertained to specific clinical areas such as general practice or anesthetics. For example, there were several instances where graduates who were anesthetists and GP anesthetists described their foundational skill development in this discipline commenced during the LIC year, and the self-efficacy they developed was reinforced in subsequent training when their more advanced skills were recognized and rewarded with further opportunities to develop.

It was incredible. And I got to do intubation, and spinal anesthetics and things where like now I’m going to be well, after next year... doing training in anesthetics. I’ll be a GP anesthetist, I think it is a large part of my experiences there (LIC program) (GP anesthetics registrar, rural work).

### Learning in place

Learning in place or the program setting was described as influential on participants’ career decisions. Place was described as the rural setting, general practice clinic, rural hospital, and specific clinical settings (e.g., operating theatre) or a combination of settings. Place-related concepts including a sense of community, comfort in the environment, and type of clinical exposure influenced participants’ decisions to work rurally and/or in general practice.

### Community

A sense of community influenced participants’ decisions to pursue general practice. The attractiveness of general practice as a specialty was centered around experiencing a friendly environment, with minimal hierarchy, where participants interacted and observed both practice staff and GPs who seemed to enjoy and derive a sense of fulfillment from their work.

Participants also acknowledged the importance of a relationship with a community of patients. A sense of personal and professional fulfillment was derived from getting to know patients and developing sustained relationships over time where they followed their journey sometimes only in general practice, but often over multiple clinical settings. This was contrasted against a less favorable preference for episodic care such as what occurs in Emergency Departments where there may be an absence of closing the loop in following patient outcomes.

So, it’s really you know your patients, sort of well and I think that’s actually what, what I enjoy probably the most about the GP is that ongoing relationship you have with patients…And sort of build that relationship with them rather than sometimes in hospitals and other specialties, you can just... you see them once or twice, and often you never really see them again (GP, metropolitan practice).

### Exposure

The exposure to general practice and participation in parallel consulting, opened participants’ eyes to the breadth of medicine within general practice, notably rural general practice. Prior to the clerkship, some participants thought they might enter general practice, but many participants who eventually became GPs described being initially unsure the specialty would be the correct choice for them, believing the clinical presentations would be of a low acuity therefore not sustaining their professional interest’s long term.

Doing GP practice, as an Immerse student I guess that was my first exposure to the wide spectrum of GP and I guess how challenging some of the patients are, and the wide variety of patients, and how interesting GP was (GP, metropolitan practice).

The breadth of exposure and type of care provided (high and low acuity) across both the general practice and hospital setting was deemed an attractive element of rural general practice. This was particularly evident in participants who have since pursued careers in rural generalism as they placed a high personal value on the additional scope of practice they observed and the value this could provide to a rural community. GPs who were not rural generalists also felt the experience awakened them to the extended skills GPs could pursue. As such undertaking specialized interests in areas such as women’s, sexual, or mental health.

And then, previous to doing rural clinical school (RCCS) GP was always a career path option but when I actually was in X [RCCS location] and got to spend time with a GP obstetrician I was like oh this is, this is actually what I want to do, I like general practice and I like obstetrics, I want to be able to do these (GP obstetrics registrar, rural practice).

The parallel consulting experience also provided formative experiences with particular patient groups such as children and patients with mental health presentations, where the available appointment time allowed for honing participants’ communication skills and strengthened their understanding of the social context of illness. This experience was acknowledged as influential in pursuing careers in medical specialties such as pediatrics and mental health.

### Comfort

Learning in place over a longitudinal period was described as providing a feeling of comfort or making participants comfortable in particular clinical settings, and/or the rural medicine environment. The development of comfort and self-efficacy in the rural environment translated to participants feeling confident to work rurally and instilled a sense of social accountability as they were contributing to increasing access to care for patients in need.

I guess it’s somewhere (rural) that I feel more comfortable with from my training, particularly. That’s probably one of the main reasons I guess that there’s, there’s a fairly significant demand and workforce shortage, so there’s not only an availability of jobs, but it feels like, a very worthwhile thing to pursue professionally in terms of meeting that public need as well (Hospital Medical Officer, rural practice).

Learning in place also contributed to participants feeling comfortable with particular groups of patients or the types of presentations they encountered within these settings. This enhanced participants’ self-efficacy and contributed to them following certain career paths.

I was quite comfortable with kids, I’d see a lot of them, examined their ears, nose, throat, yeah play with them, just mucking around with kids in the GP land … so you’re chatting to them, you’re building rapport, you’re examining them it’s that sort of exposure that you wouldn’t really get in a pediatric ward. So, I think yeah there’s a level of comfort in seeing children is definitely IMMERSE related for sure (Pediatrician, rural practice)”.

Some participants contrasted their comfort level in the smaller rural clinical settings with their aversion to, or discomfort in, larger tertiary hospitals which stemmed from factors such as perceived lack of support, absence of a sense of community, and preference for rural living. Participants who valued living rurally and were more comfortable in this environment described selecting their specialty based on what training could be completed entirely rurally.

## Discussion

This constructivist grounded theory study explored how participating in a rural LIC influenced medical graduates’ career decisions in terms of both medical specialty and geographic practice location. The findings add to the existing quantitative workforce data associated with rural LICs and highlight the contextual program elements that influence graduates’ workforce decisions. Key concepts confirm previous findings that participation, rather than career intention is a key influence on LIC medical students’ career decisions [24]. Participation had a transformative capacity for all students, those who self-selected into the program and those who did not.

A holistic view of workforce decisions aligned with the LIC is theorized and not limited to elements that only influence rural or primary care careers. As such, not all aspects of the theoretical framework symbiotically, unidirectionally, and systematically link together to produce workforce outcomes, but in essence it illustrates an ecological theoretical framework where there is a dynamic interplay between elements [25]. For example, learning in-place underpinned by a sense of community may have influenced a graduate’s decision to pursue general practice, but not necessarily within a rural location. Or learning design affordance may have influenced a career in anesthetics due to the interplay between the autonomy experienced, personal and professional compatibility with the specialty, and self-efficacy from the development of skills.

However, the framework does support that a combination of learning in place and learning design affordance introduced and/or confirmed participants’ pursuit of rural general practice [8, 10, 26]. Such findings may not seem surprising, but the presentation of elements that support this outcome and demonstration of ‘what works’ has the potential to inform future LIC program development [16, 18]. Barrett et al. (2020) developed guidelines that state the intended goal of workforce transformation should be explicitly stated within LICs as they are not always intrinsically interwoven into every LIC [27]. Alignment of the specific program goals with the influential program elements may have the capacity to enhance the intended outcomes.

Previously, studies have found having a GP as a role model and positive exposure to general practice in the curriculum are significant influences on pursuing general practice careers [28, 29]. Both these influences were present in our study and were facilitated by elements of the framework, in particular compatibility, comfort, and community that allowed the student to gain a realistic authentic exposure to general practice. Globally, as fewer medical students are pursuing primary care [30, 31], it is arguable that all medical students, not just those in an LIC, should be provided with longitudinal exposure to primary care. The findings from this study illustrate a community of practice where a group of people worked towards common goals, resulting in the formation of a common identity and a sense of belonging [27, 32].

During the LIC year, students move from the periphery to the center of a community of practice, transforming into an authentic team member [32, 33]. This period of transformation is formative and has been found to influence professional identity formation (PIF) [18, 27, 32]. PIF is a complex, dynamic, and constructive process that the individual employs to link motivations and competencies to a chosen career [33, 34]. Reflection is integral to both PIF and transformative learning, with the latter being described as a cornerstone of the LIC experience [19, 35]. During any given day/week LIC students participate in a variety of clinical settings and as such a continuous active and passive reflection process occurs. Practically the LIC students in this study were able to compare and reflect on medical specialties simultaneously, as opposed to retrospective assessment after each discipline-specific block rotation which may have the capacity to reframe experiences and introduce recency bias. The autonomy of the program facilitated this simultaneous reflection and comparison, with participants drawn to and spending more time in particular clinical areas, where they felt their personal and professional values aligned. In turn, this led to skill proficiency, self-efficacy, and feeling comfortable and was often indicative of the vocational pathways graduates eventually pursued.

Previous studies have hypothesized that students who are interested in primary care or rural medicine may self-select into rural LICs based on the program’s alignment with their personal values and career intent [36, 37]. While this was evident, a unique finding from our study was that non-self-selectors also experienced a transformation with pre-conceived career intentions challenged and transformed. This was particularly apparent in relation to rural medicine and general practice where pre-existing negative perceptions were challenged and reframed to become positive. This aligns with recent research that found medical students’ views of general practice are often influenced by external factors such as negative comments by others and a perception that it is not intellectually challenging [13]. Integral to this transformation is the relationship with and role modeling of supervising GPs who can have a powerful influence on students, but there is a need to balance this influence as too much focus on trying to ‘push’ students into rural or general practice and unsolicited career advice has been described as having a negative influence [11, 13]. Due to the importance of this student/supervisor relationship, universities should strive to recruit supervisors aligned with their program’s goals and ensure they are adequately supported with real-time feedback and ongoing training [19, 38].

### Limitations

Interviews were undertaken with medical practitioners who were between two- and eleven-years post LIC experience, as such their reflections may be influenced by the passage of time. However, this period addresses a gap in the literature as most participants had either completed their medical specialty training or had joined a medical specialty training pathway therefore findings were based on actual career outcomes rather than career intention.

## Conclusion

The developed framework postulates how participation in the rural LIC could transform and influence graduates’ future workforce decisions. Transformation occurs through reflection, the learning design affordance, and learning in place which can influence professional identity formation. Overtly identifying these key components of the LIC experience provides an opportunity to further impact rural workforce outcomes, by strengthening these elements in new and existing LIC programs and exploring the transferability of this framework to other medical education models.

## Data Availability

The datasets generated and/or analysed during the current study are available from the corresponding author on reasonable request.
